# COVID-19 and Mucormycosis of Orofacial Region: A Scoping Review

**DOI:** 10.7759/cureus.37984

**Published:** 2023-04-22

**Authors:** Abhisek Banerjee, Moumalini Das, Pooja Verma, Abhishek Chatterjee, Karthikeyan Ramalingam, Kumar Chandan Srivastava

**Affiliations:** 1 Oral and Maxillofacial Pathology, Awadh Dental College and Hospital, Jamshedpur, IND; 2 Dentistry, Rampurhat Government Medical College and Hospital, Rampurhat, IND; 3 Oral pathology, Saveetha Dental College and Hospitals, Saveetha Institute of Medical and Technical Sciences, Chennai, IND; 4 Oral and Maxillofacial Surgery, College of Dentistry, Jouf University, Sakaka, SAU

**Keywords:** treatment, investigations, diabetes, clinical presentation, diagnosis, pathogenesis, rhino-orbital cerebral mucormycosis, mucormycosis, sars-cov-2, covid-19

## Abstract

During the second wave of coronavirus disease, or COVID-19, infection due to severe acute respiratory syndrome coronavirus 2 (SARS-CoV-2) virus in the year 2021 around the globe, there is a surge in the number of cases of mucormycosis or “Black Fungus” that is directly/indirectly associated with COVID-19.

In this review article, mucormycosis of the orofacial region has gained importance from the maximum published literature (45 articles) from various databases like PubMed, Google Scholar, Scopus, Web of Science, and Embase.

Rhino-orbital cerebral mucormycosis (ROCM) is a fatal condition associated with COVID-19 among categories of mucormycosis such as pulmonary, oral, gastrointestinal, cutaneous, and disseminated. ROCM targets the maxillary sinus, also involving teeth of the maxilla, orbits, and ethmoidal sinus. These are of particular interest to dentists and oral pathologists for proper diagnosis and identification. Co-morbid conditions, especially diabetes mellitus type II, have to be monitored carefully in COVID-19 patients as they have a higher risk of developing mucormycosis. In this review article, various presentations of COVID-19-linked mucormycosis are mentioned having particular emphasis on pathogenesis, signs and symptoms, clinical presentation, various diagnostic modalities including histopathology, radiology like CT and MRI, serology, tissue culture, various laboratory investigations, treatment protocols, management with prognosis, and so on. Any suspected case of mucormycosis needs quick detection and treatment since it progresses quickly due to the destructive course of infection.

Long-term follow-up along with proper care is a must to detect any kind of recurrence.

## Introduction and background

The coronavirus SARS-CoV2 (severe acute respiratory syndrome coronavirus 2) virus (enveloped RNA virus) resulted in a massive outbreak of COVID-19 in 2019, starting in China and then spreading all over India as well as other parts of the globe. It continued for two years with the first, second, and third waves until now. It was declared a pandemic by the World Health Organization. It resulted in acute respiratory distress syndrome (ARDS) in a maximum number of patients [1].

The launch and global distribution of vaccinations like Covishield and Covaxin at a rapid pace is a great relief for mankind and a ray of new hope to combat the disease [2].

COVID-19 pandemic’s second wave gifted a superinfection named mucormycosis or “Black Fungus,” an opportunistic fungal infection. COVID-19 is related to various devious co-infections both from bacterial and fungal origin. The trinity of typical co-infections arising secondarily of fungal origin of the oral cavity included candidosis, mucormycosis, and aspergillosis [1].

Mucormycosis became the second most popular interfering mold septicemia and is an epidemic within a pandemic. COVID-19-associated mucormycosis (CAM) is very challenging, as it was tough worldwide to sway the rapidly mutating COVID virus and also for less knowledge to treat mucormycosis as it is considered an infrequent infection [3]. Untreated mucormycosis is rapidly fatal, having high morbidity and mortality, and the term was first coined by Baker in 1957 [4].

Mucormycosis resulted from different fungi species, mainly Mucorales. Mucorales spores have their existence widely in nature in the mucosa of the nose of individuals as a normal commensal. It is uncommon, feeds on dead and decaying organic matter, is opportunistic and deadly, and results from phylum Zygomycota fungus, subphylum Mucormycotina of the Mucorales group [3]. It buds as a pathogen within the paranasal sinus and grows into orbit or intracranially also if the patient is immunocompromised [2].

Candida organisms transform into an opportunistic fungus with altered situations, e.g. COVID, resulting in the local or systemic spread. Isolated COVID-19 infection or associated co-existing factors like corticosteroid treatment, decreased blood lymphocyte number, patient under ventilation, and other local conditions, e.g. poor oral hygiene, xerostomia, and patients wearing dentures resulted in Candida infection in the mouth. Mucormycosis (termed as zygomycosis earlier) is an angio-invasive illness. Upon six well-recognized clinical subtypes, the variety/structure of mucormycosis is as follows - rhino-orbital cerebral mucormycosis (ROCM), cutaneous, pulmonary, gastrointestinal, disseminated, and others. ROCM is popular most frequently in patients having less immunity, whereas cutaneous mucormycosis is prevalent in patients with good immune responses. The third most common variety of mucormycosis is the cutaneous one [3].

Mucor and Rhizopus species are the most common mucormycosis causative organisms. They are seldom found in the oral cavity normally and get transmitted generally by inhaling fungal spores from air and dirt. In Western Countries like the US, and Europe, they occur in 0.01-0.02 per 100,000 citizens. India shows a rate of 14 per 100,000. In adults, the mortality is between 20% and 100% for other factors like comorbidities, infection site, and timely therapeutic interventions, with emphasis on the system of healthcare providers. In children, 33.3% is the range of mortality [2].

According to different studies, the etiology for Mucorales growth in patients having SARS-CoV-2 is hypoxia, diabetes, hyperglycemia caused by steroids and ketoacidosis, low immunity, long hospital and intensive care unit (ICU) stays, ventilator support, and so on in India, and there are reports showing mucormycosis of the orofacial region in SARS-CoV-2-infected patients with devastating presentations even leading to blindness. Among the COVID-19-related trinity, the final one is aspergillosis of common oral and maxillofacial fungal infection. Fifteen percent of cases showed that patients getting admitted to hospitals with SARS-CoV-2 infection in the ICU had *Aspergillus* infection. The main target is immunocompromised patients having diabetes, undergoing stem cell and organ transplantation, and having hematological malignancies. Laboratory diagnosis and treatment in COVID-19 patients with mucormycosis are the challenges [3-5].

This study aims to review cases of orofacial mucormycosis associated with COVID-19 either occurring directly or as a secondary factor during the COVID-19 outbreak. The etiopathogenesis, clinical cases, associated factors, prevalence, and oral and maxillofacial clinical manifestations and treatment are described and reported in various cases. Herein, we tried to interrelate COVID-19 and mucormycosis. The maximum of cases have been tried to be included as per articles searched from different journals, for spreading awareness, diagnosis, and proper information among individuals, the medical and dental fraternity, and the common public.

## Review

Search strategy

We collected data from 45 articles published mainly in PubMed, Scopus, Google Scholar, Web of Science, and Embase typing keywords like COVID-19, oral mucormycosis, black fungus, and oral and maxillofacial manifestations. Review articles, case reports, and letters to the editor are presented in this review article.

**Figure 1:**
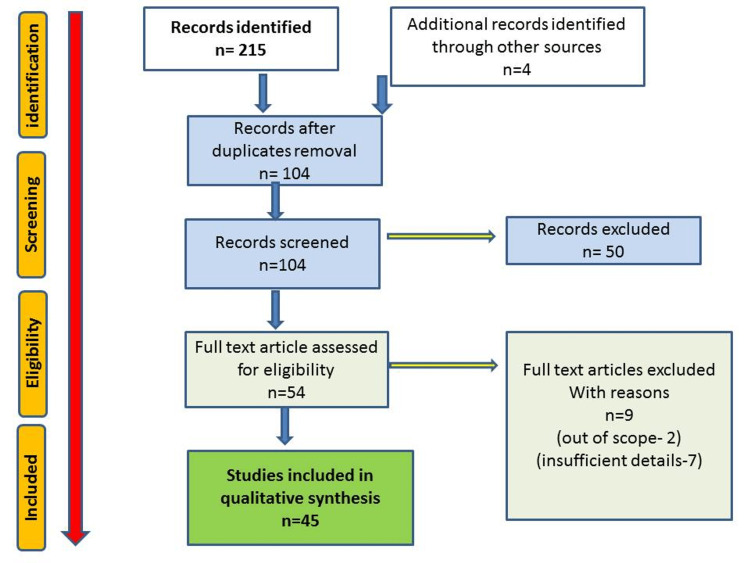
Search Strategy Flow chart showing the search strategy and analysis of articles.

A Preferred Reporting Item for Systematic Reviews and Meta-Analyses (PRISMA) protocol has been adopted (Figure 1).

Pathophysiology

COVID-19 enters the cell through receptors namely angiotensin-converting enzyme 2 (ACE2) and trans-membrane protease serine 2 (TMPRSS-2). ACE2-R has higher expressivity in the respiratory, kidney, and digestive tract epithelium. Receptors of TMPRSS-2 are located particularly in the respiratory and gastrointestinal epithelium among many epithelia. It tends to attack lymphocytes when binding with these cellular ACE2 receptors, inducing lymphopenia, lowering counts of CD4+ and CD8+ T-cells, and therefore depressing the immune system [2,4].

This destruction happens due to increased interleukin measures (interleukin-2,6,7, interferon-gamma inducible factor, granulocyte colony-stimulating factor) inducing cytokine storm. This results in the following:

1. deterioration of lymphoid tissue;

2. compromised defense system;

3. decreasing future production;

4. increased protective lymphocytes [2].

Also, the effect of lactic acidosis knocks down an alveolar type 2 cell and affects the rebuilding of bronchial cells, consequently causing respiratory difficulties. Therefore, increasing acid-base levels finally results in hypoxemia and hypoperfusion. Since the cytokine storm needs to be immediately treated by immunosuppressive steroids, an optimal environment is generated for fungal growth. Finally, two other conditions give energy booster to the increase of Mucorales in the body of infected individuals:

1. Ferritin levels get shot as there is a higher breakdown of red blood cells (RBCs) (iron is deadly to phagocytes).

2. Increased body temperature (these organisms can tolerate high temperatures).

Mucorales get their nourishment as a result of destruction by ACE-2 receptors to beta cells of the pancreas causing higher glucose levels of plasma. So, mucormycosis is more prevalent in diabetic patients [2,3].

Mucormycetes enter blood vessels through damage to the endothelium, insulin opposition, and elevated levels of glucose leading to the growth of the Mucorales, and continuous destruction of the already damaged immunity of the patient. So, it leads to the eventual deterioration of the patient [2].

The fungi being bigger in comparison to others get easy access to the paranasal sinuses. But small-sized species may also be found in the lungs.

The most important process in the pathogenesis of mucormycosis is as follows:

Angioinvasion by Mucorales fungi results in embolism of the affected vascular supply, therefore resulting in tissue ischemia and necrosis.

Less blood supply as a result of blockage of blood vessels by Mucorales protects it by resisting systemic antifungal drugs and the host’s defense system from approaching the infection site [6].

For thrombosis to occur, the fungi require adhering to endothelial cells and destruction of cohesion entering the bloodstream [6-8]. 

Role of iron

*Rhizopus oryzae* requires ferrous/ferric for main cellular activities for cell advancement with evolution. In a normal way, the serum of the mammalian body contains iron in bounded forms like transferrin, ferritin, and lactoferrin. These protect from toxicity resulting from free iron. The human body’s free iron introduction is very much needed to uplift *R. oryzae* by distinct mechanisms. They naturally discharge iron chelators of low molecular weight and are termed “siderophores,” which are iron lovers. *R. oryzae* produces polycarboxylate siderophore “rhizoferrin” and gets iron from the body of the host by an energy-dependent receptor-moderated process. But, rhizoferrin is not able to draw out iron from the serum of the host. Endogenous siderophores have a limited role in its pathogenicity [5,6].

In renal dialysis patients, due to excess iron production, deferoxamine is included during treatment, since they have a chance of developing toxicity. They chelate directly bound iron in transferrin, forming an iron-ferrioxamine complex. The ferrous iron gets free by depletion at the surface of the cell of Mucorales by Fob receptors [6,7].

*Rhizopus* spp. is 8-40 times the quantity of iron by deferoxamine. Free iron obtainability depends upon the seriousness of the disease caused by *Rhizopus* spp. Conditions like acidosis decreasing the iron-adhering capability of transferrin through proton-modulated substitution of ferric ions create beneficial environments for Mucorales growth. In patients having continued blood transfusions as a result of diseases like myelodysplastic syndrome, iron overload is caused ultimately causing mucormycosis [6].

Risk factors could be local or systemic in nature.

Local Factors

Inhaler usage, sinusitis of acute origin, injection, trauma, or burns can be considered.

Systemic Factors

Diabetes mellitus (uncontrolled) is one of the major causative factors.

Decreased immunity, drug use, hematological disorders or diseases, malignancy-related disorders, hematopoietic stem cell transplantation, and solid organ transplantation [5,6] are other important factors. Uncontrolled diabetes is likely to be one of the most important causative factors to produce mucormycosis in developing countries like India.

Environmental Factors and Causes of COVID-Associated Mucorrmycosis (CAM)

**Figure 2:**
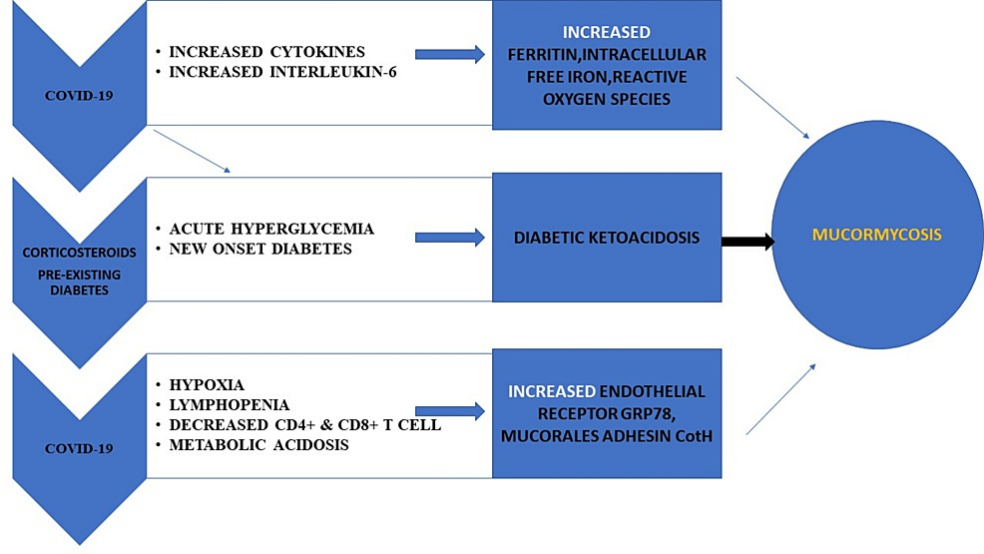
Causes and pathogenesis Graphical representation of causes and pathogenetic mechanisms of mucormycosis.

Humans acquire mucormycosis disease (universal distribution) through inhalation, consumption, or traumatic infusion of the sporangiospores of Mucorales fungi from the atmosphere. But the spore load is higher in tropical countries. Mucorales spores are found in the air in India’s indoor and outdoor environments. *Rhizopus arrhizus*, the major pathogenic species, is also the predominant species isolated from the environment [7]. Diabetes mellitus is a significant consideration for the spread of mucormycosis infection In COVID-19 patients (Figure 2).

Passages of Access

Although they are universally present, these fungi are rarely found as normal commensal microflora of the dermis/abdominal tract.

Mucorales are not capable of permeating by undamaged epidermis/dermis or mucosa. Break in the mucocutaneous cohesion due to injury/tear and laceration/mechanical procedures/extraction sockets of teeth creates passages for fungal entry. Also, the Mucorales spores get access through the nose or get direct entry into the oral cavity by using uncleaned objects and hospital materials, etc., in dental setups like forceps, burs, or airotor handpiece [6].

The most common mode of entry is the ventilators (mechanical) giving oxygen to hypoxemic patients. The possibility of entry can be during drug delivery in the treatment of COVID-19/easy entry into the blood circulation by catheters used intravenously/subcutaneous injections [5,6,8].

Mucormycosis diagnosis [8]

Diagnosis

It can be done by diagnosing Mucorales from the affected tissue by performing a biopsy and thereafter histopathology. A validatory fungal culture in microbiology is also important. Diagnostic imaging techniques, Molecular Assays, and Direct identification of fungus from the body’s secretions like blood, plasma/serum, pleural fluid, and urine are equally significant.

Diagnosis Based on Clinical Approach

Identification of host-microbial interaction along with its varied clinical presentation is important for prompt response.

Double vision, exophthalmos, discomfort in the sinus, periorbital edema, palsy of the cranial nerve, palatal ulceration, and apex syndrome of the orbit are the “RED FLAGS” [8].

Based on Smith and Kirchner criteria, the gold standard to confirm mucormycosis, the diagnosis clinically comprises the following:

a. If dried and crusted blood is present, black and necrotic turbinates may be misdiagnosed.

b. Nasal blood-colored secretions are linked to facial pain in the affected region.

c. Soft swelling with darkening around the peri-orbital or peri-nasal area that is related to the development of induration.

d. Abnormally low-positioned upper eyelid with protrusion of the eyeball from the orbit, total oculomotor paralysis.

e. Multiple paralyses of the cranial nerves [9].

Microscopic examination and culture

Direct Microscopy

It is done with biopsied samples with the help of optical brighteners like Blankophor and Calcofluor White, which aids in fast diagnosis. Fungal hyphae, whether non-septate/pauci-septate, differ in dimensions and have ribbon-like features and they are uneven. They are distinctly seen on periodic acid-Schiff (PAS stain), H&E stained sections, Grocott-methenamine, and Gomori’s silver stains.

Histopathology

Inflammation (neutrophilic/granulomatous) and invasive lesions are differentiated since they have angioinvasion and large closure. Perineural invasion is visible in cases where nerve structures are involved.

Necrotic tissue with inflammatory constituents is found, namely neutrophils, lymphocytes, macrophages, and giant cells. Inflamed granulation tissue, hemorrhagic elements, and extravasated RBCs are also noticed. The necrotic tissue shows the appearance of broad, non-septate hyphal forms [9].

Tissue Culture

Sabouraud agar and potato dextrose agar were used. They are cultured at (25°C to 30°C). In them, every fungus, especially Mucorales, develops fast (3-7 days). In half of the cases, results are positive for Mucorales.

Immunohistochemistry

A certain monoclonal anti-Rhizomucor antibody of the mouse is used [8].

Antifungal Susceptibility Testing and Identification of Species

Mucorales fungi are easily differentiated from Aspergillus during culture. On inspection by specialists in the fungal field, morphological characteristics might help in prompt diagnosis. ID32C kit (bio Merieux, Marcy lÉtoile, France) is used for identifying *Rhizomucor pusillus* and *Lichtheimia corymbifera* and API 50CH (bioMerieux, Marcy-l’Étoile, France) for Mucor species [8].

Serological Examination

ELISA (enzyme-linked immunosorbent assay) technique, Western blot tests, and Ouchterlony tests (allows antigen detection) are among the most important ones.

Tests Using Molecules

Polymerase chain reaction (PCR) performed routinely, chaining of DNA of particular genes, melt curve investigation of products of PCR, and restriction fragment length polymorphism analyses are very significant.

All of the assays mentioned above can be used to detect or identify Mucorales for internal transcribed spacer or the 18S rRNA genes [8].

Imaging

CT scanning of both sinus and lungs: Partial or complete cloudiness para-nasal sinuses whether single or multiple, division line appearance between healthy and necrotic bone, also associated teeth movability, and the emergence of sequestrum in maxillary/zygomatic bone are seen; the orbit is also not spared with prominent soft tissue and of adipose tissue dissolution in and surrounding pterygopalatine fossa.

Chest CT scans imaging: Cavities, nodules, pleural effusion, halo signals, and wedge-shaped shadows are the changes seen. Blood vessel capture is also imaged, which denotes a sort of fungal infection but mucormycosis is not specified.

Patients having reduced neutrophil counts and blood cancer show a reverse halo sign (RHS) indicating mucormycosis.

MRI and MRI with contrast agent gadolinium are used in ROCM (method of choice).

MRI signs:

a) Demonstration of "black turbinate" indication on axial/coronal slices is classic for rhinosinusitis of fungus.

b) Lesions of the sinus (non-enhancing) and outside sinus.

c) Angioinvasion and fungal vasculitis cause thrombosis of the internal carotid artery, retina’s central artery, and arteries of the cerebrum.

d) Contrast-enhanced (CE) scans are used in places of debilitated tissues within and surrounding the ethmoidal, orbit, and maxillary sinuses.

e) Cavernous sinus embolisms are found in the non-enhanced lesion on a CE fat-saturated MR picture.

f) Extension appears as an intracranial hypointense dural amplifying infection.

Dental and Oral Presentations

The manifestations are as follows:

Toothache, teeth mobility, foul- or bad-breath-causing halitosis, blockage in the nose, epistaxis and nasal ejection, black pus release, necrosis of bone/sequestrum development in palatal bone and alveolar bone of maxilla, oro-antral or oro-nasal communications or fistulae, pain in and around sinus, trismus-affecting muscles of mastication, unhealed extraction sockets having characteristics same as alveolar osteitis/chronic osteomyelitis, ulceration in the palate, erythematous face, skin getting discolored (black), draining sinuses intra and extra orally, nasal mucosa getting erythematous, erythema around the orbit, swelling and inflammation of subcutaneous connective tissue, the pain of orbit, drooping upper eyelid, double vision, blindness, paralysis of motor nerves of the eye, and migraine [8].

Tables 1-3 of Appendices depict the summary of our review findings [9-38].

Management [39-42]

There are different surgery protocols, antifungal treatment and follow-up, monitoring, and other advisories from different associations or institutes as follows:

1. All India Institute of Medical Sciences

2. Clinical Infectious Disease Society

3. Directorate General of Health Services-India

4. European Confederation of Medical Mycology and the International Society for Human and Animal Mycology

5. Indian Council for Medical Research

6. Indian Medical Association.

These protocols of surgery and treatment options are not discussed in detail.

Surgical Treatment

a. Turbinectomy

b. Debridement of sinuses of maxillary, ethmoidal, and frontal 

c. Maxillectomy, palatal resection

d. Orbital exenteration [39,40].

Complications

It results from the disease or can be the management or therapies of mucormycosis.

a. From the disease: Mucormycosis arising from maxillary sinuses in diabetes mellitus patients usually involves the rhino-cerebral region.

b. Angioinvasion is the hallmark of mucormycosis [7,8].

c. Coverage of mucormycosis includes orbit-causing ophthalmoplegia and lessening of visual perceptivity. Complete blindness results from thrombosis of the central artery of the retina.

d. Mucormycosis may reach out in a posterior direction from the sinus of the maxilla and cavity of the nose even to the infratemporal fossa, pterygopalatine fossa, and masticatory muscles resulting in trismus and reduction of mouth opening [7-9].

e. Disturbances of the gastrointestinal tract, toxicity of the liver, kidney, and heart, infusion-related complications, swollen face, allergy, sensorineural deafness, achromatic vision, migraine, fever, anorexia, loss of calories, disturbances of electrolytes, and creation of advanced mold septicemia are few reported complications of different medicines utilized in the therapies of ROCM [41-43].

f. Complications after surgeries are problems in chewing, speaking, and nasal backward flow of fluids in case of a palatal defect. Facial, nasal, and oral deformities can occur during surgical treatment of ROCM [8,44].

Prevention

a. Patients with blocked noses should be considered in cases of poor immunity and/or COVID-19 patients on immunomodulators.

b. Early characteristics of mucormycosis should be advised to patients during discharge.

c. Normal tests such as pupillary reaction, ocular motility, sinus tenderness, and palatal inspection are done for regular clinical checkups of COVID-19 patients [35-38,45].

Finally, proper control of infections by dental surgeons/oral pathologists/oral radiologists/technicians is a must and they should follow everything according to new guidelines in a COVID-19 scenario from handling a patient to various dental procedures/radiological procedures whether in a dental setup or academic institute [46].

All healthcare professionals including those >40 years (having greater experience and Master’s degree) have good knowledge regarding protocols to be followed at the time of COVID-19 including the use of personal protective equipment (PPE), travel restriction protocols, and all other information regarding COVID in India and Saudi Arabia. Still, further, continuing education programs are necessary [47].

Allied healthcare professionals (AHPs) must also develop further knowledge and keep a positive attitude to increase their skills about everything in the COVID-19 scenario, and in the private sector, strategies must be enforced for better performance of AHPs. This must be done in all countries including India and Saudi Arabia [48].

Prognosis

Prognosis depends on basically the extension of spread, aggressiveness, time of diagnosis and treatment, and immune status (especially the presence of any hematological diseases). The localized infection has a superior prognosis. Extension intracranially has a bad prognosis [40,45]. When there is no systemic ailment for rhino-cerebral disease, there is a 75% rate of survival.

ROCM (with systemic illness) has a 20% survival rate [6,8]. Diagnosis of ROCM can be done very advance than pulmonary mucormycosis. Therefore, ROCM with early detection and therapy has a better prognosis and survival rate. In total, reported mortality with all types of mucormycosis is 40%-80%. The chances of living are poorer in victims having malignancies of hematological origin and transplantation of any organ. The recommendation for follow-up is 36 months as there is a 13% recurrence in three-year postoperative patients. In a follow-up of five years as a whole, the rate of survival is 60% [8,45].

Social media played a good as well as bitter role during the COVID-19 outbreak. It was used for social awareness and it also created mass hysteria. Hence, social media platforms are a double-edged sword that has to be handled with government monitoring [49].

Several opportunistic infections were reported in COVID-19 patients, including *Aspergillus* spp., *Candida* spp., *Cryptococcus neoformans*, *Pneumocystis jiroveci* (carinii), mucormycosis, cytomegalovirus (CMV), herpes simplex virus (HSV), *Strongyloides stercoralis*, *Mycobacterium tuberculosis*, and *Toxoplasma gondii* [50]. The estimated prevalence of mucormycosis is approximately 70 times higher in India than in the rest of the world [51]. A total of 388 proven/probable mucormycosis cases were reported during the study period with overall mortality at 46.7%. The mortality rate was significantly higher in north Indian patients (50.5%) compared to 32.1% in south India (P = 0.016). The study highlights a higher number of mucormycosis cases in uncontrolled diabetics of north India and the emergence of *R. microsporus *and *R. homothallicus* across India causing the disease [52,53]. 

Clinical significance

Mucormycosis/“Black Fungus” is a fatal fungal infection that is associated with COVID-19 and came as an epidemic within the pandemic in the second wave of the infection. Mucorales fungi cause angioinvasion resulting in thrombosis of affected blood vessels and thereafter tissue ischemia and necrosis. Among various subtypes, rhino-orbital-cerebral mucormycosis is very dangerous and found in maximum cases of COVID-related lesions. This superinfection having a high mortality rate is seen especially in patients having comorbid conditions, particularly diabetes mellitus type II, immunosuppression states, and prolonged ICU stay during COVID-19 treatment. Black purulent discharge with palatal ulceration, bone necrosis, formation of sequestrum in the palatal region, and development of OAF (oro-antral fistula), oro-nasal or (OAC) oro-antral communications or maybe fistulae, non-recovered extraction sockets, draining sinuses, difficulty in mouth opening, etc. are some of the clinical presentations. As an oral pathologist, the sole purpose is the earliest detection and diagnosis of risk factors so that they can be managed whether by surgery or an antifungal regimen. Patients having recovered from COVID-19 must be explained about mucormycosis and to seek a dentist’s help immediately in any sort of such clinical manifestations. The treatment plan is to be very carefully decided, with long-term follow-up to avoid chances of recurrences and for a better prognosis.

## Conclusions

COVID-19 and its association with mucormycosis can be a serious problem in the scenario of COVID-19 infection, especially in the second wave. There is a boost in mucormycosis cases in the COVID scenario. Oral and maxillofacial fungi, if exist, appeared jointly along with COVID symptoms or maybe in a straightaway healing phase. These are most commonly and frequently seen in patients having comorbid conditions, especially type II diabetes mellitus. 

Patients infected with COVID-19 are prone to develop oral fungal opportunistic infections. Etiology can be many, under which decreased immunity as a result of latent viral infectivity, immunosuppression, and treatment of steroids for COVID, ventilator-supplemented Mucorales growth, xerostomia, and diabetes can be given importance. This was also found in patients who recovered from COVID-19 and prolonged ICU patients. They may have a certain degree of immunosuppression.

The research on the complex relationship between SARS-CoV-2 and mucormycosis is ongoing, as it is not properly established. ROCM is a serious condition and must be detected early and properly treated. It is a challenging fungal infection plus its mortality rate in COVID patients is towering, mainly in patients with pulmonary disease.

If attentive therapeutic planning is advocated, the rhino-orbital cerebral disease can be successfully managed. Faster recognition with control of CAM must be done properly; otherwise, they can be fatal. Dental surgeons and oral pathologists must detect and identify the symptoms, risk factors, and clinical manifestations at the earliest with proper investigations and treatment for a better prognosis.

## Appendices

**Table 1:** Features of oro-facial mucormycosis Table representing oral and maxillofacial features of mucormycosis.

Study conducted by	Mucormycosis (no. of patients)	Patients admitted in hospital	Hospital stay	Signs and symptoms	Mucormycosis presentations (commencement)	Mucormycosis manifestations
Pauli MA et al. (2021) [10]	Oral mucormycosis - asymptomatic/mild COVID-19 cases (n = 1)	Quarantined patient	1.5 months	An uncomfortable, painful deep ulcer on the hard palate (near midline)	1 week post-COVID manifestations	Hard palate
Diwakar J et al. (2021) [11]	Maxillofacial mucormycosis - mild COVID-19 cases (n = 2)	Ambulatory long COVID patients having hyperglycemia and diabetic ketoacidosis	-	Case 1 - swelling with pain of an eye from 1 week, and very high fever up to 3 days; Case 2 - pain, edema, and double vision	During COVID-19 diagnosis	Rhino-orbital cerebral mucormycosis (ROCM)
Revannavar SM et al. (2021) [12]	(n = 1)	Ambulatory COVID-19 patient with type 2 diabetes	-	Complete ptosis, fever for few days	During COVID detection	Acute infarct in the left parieto-occipital region, pansinusitis on the left side, and oculomotor paralysis
Saldanha M et al. (2021) [13]	(n = 1)	Ambulatory COVID-19 patients and (uncontrolled) diabetes	-	Left eye ptosis, pain in face - 5 days	During COVID-19 detection	Nose, paranasal sinus, orbital apex mucormycosis
Alekseyev K et al. (2021) [14]	Oral mucormycosis in moderate/severe COVID-19 cases (n = 1)	Hospitalized	-	Loss of taste, deep-aching pain in the nose that radiates down to throat, dry cough	Same time with COVID identification	Black wound - hard palate
Ashour MM et al. (2021) [15]	(n = 6)	Hospitalized	14 days-1 month	Symptoms to COVID-19 infection	Invasive fungal disease 12-35 days from starting symptoms	6 patients - there was involvement of hard palate
Riad A et al. (2021b) [16]	(Of n = 7, 1 patient has palatal mucormycosis)	Hospitalized	-	Painful lesion - hard palate for 4 days and also fever, neurological symptoms	After getting better from COVID-19	Case 1: Lesion - ulcer and necrosis in left side - hard palate
Veisi A et al. (2021) [17]	(n = 2)	Hospitalized	-	COVID-19 symptoms	1 week after COVID-19	Case 1: tissue that is black necrotic - in the hard palate
Waizel Haiat S et al. (2021) [18]	(n = 1)	Hospitalized	-	Left midface and lid edema with pain that extends to the upper lip and malar region	Simultaneous with COVID-19 diagnosis	Pallor - mucosa of hard palate
Werthman-Ehrenreich A et al. (2021) [19]	(n = 1)	Hospitalized	26 days	Altered mind, left eye blepharoptosis, absence of fever, heart rate increased mildly, hypertension, fast breathing	With COVID-19 detection	Dryness of oral mucosa and brown, dry secretion on palate
Bayram N et al. (2021) [20]	Maxillofacial mucormycosis in moderate-to-severe cases of COVID-19 (n = 11)	Hospitalized	6 weeks	COVID-19 symptoms	After 12-16 days from COVID-19 infection	7 cases of rhino-orbital mucormycosis, 3 cases of rhino-orbital-cerebral mucormycosis (ROCM)

**Table 2:** Features of COVID-19-associated mucormycosis COVID-19-associated mucormycosis with risk factors, types, histopathology, and clinical presentation

Author, year, study location	Underlying conditions/risk factors	Mechanical ventilation, n (%)	Use of systemic corticosteroid therapy	Histopathologic diagnosis	Mucormycosis types	Clinical presentation	Elaboration and causes
Alekseyev et al. (2021), United States [14]	Uncontrolled diabetes, diabetic ketoacidosis	No	Yes	-	Putative	Infratemporal abscess with cavernous sinus augmentation and peripheral bilateral lung infiltrates that extend into the sinuses	Rhino-cerebral mucormycosis (ROCM)/Mucorale (unspecified)
Bellanger et al. (2021), France [21]	Lymphoma, hematopoietic stem cell transplantation, under steroids for COVID	Yes	Yes	-	Putative	Pulmonary fibrosis with non-specific bilateral ground glass opacities	Pulmonary mucormycosis/*Rhizopus microsporus*
Dallalzadeh et al. (2021), United States [22]	Uncontrolled diabetes (n = 2), diabetic ketoacidosis	-	Yes (n = 2)	No	Definite	Right sinonasal cavity and anterior skull base	Rhino-orbital mucormycosis
Garg et al. (2021), India [23]	Uncontrolled diabetes, hypertension, coronary artery disease, cardiac problems, kidney disease, under steroids for COVID	Yes	Yes	No	Putative	Cough, expectoration, micturition	Pulmonary mucormycosis/*Rhizopus microsporus*
Hanley et al. (2020), United Kingdom [24]	Pancreatitis, steroid for COVID infections	Yes	Yes	Yes	Definite (post-mortem)	-	Disseminated (involving the heart, brain, kidney, and hilar lymph nodes)/Mucorale (unspecified)
Johnson et al. (2021), United States [25]	Diabetes, hypertension, under steroid for COVID	Yes	Yes	Yes	Probable	Ground glass opacities bilaterally and infiltrates; extensive bilateral pneumonia	Pulmonary mucormycosis/*Rhizopus arrhizus*
Kanwar et al. (2021), United States [26]	Last-stage kidney ailment (hemodialysis)	Yes	Yes	Yes	Definite	Pleural effusion infiltrates patchy ground glass	Pulmonary mucormycosis/*Rhizopus azygosporus*
Karimi‐Galougahi et al. (2021), Iran [27]	Diabetes, steroid for COVID	0 (0)	Yes	Yes	Definite	Right hemifacial pain having numb sensation, visual acuity gets decreased, chemosis, proptosis, frozen eye, vision lost completely etc.	Rhino-orbital mucormycosis/Mucorale
Khatri et al. (2021), United States [28]	Diabetes, hypertension, , kidney failure, solid organ transplantation, coronary artery disease, OSA	Yes	Yes	Yes	Definite	Purplish skin discoloration with fluctuant swelling of right axilla	Cutaneous mucormycosis/*Rhizopus microsporus*
Maini et al. (2021), India [29]	Steroid for COVID infection	No	Yes	Yes	Definite	Left eye pain and conjunctival edema	Sino-orbital mucormycosis/*Rhizopus oryzae*
Mehta et al. (2020), India [30]	Diabetes (uncontrolled) steroid for COVID	1 (100)	Yes	Yes	Definite	Facial swelling on one side, periorbital facial pain unilaterally, eyelid edema, etc.	Rhino-orbital-cerebral mucormycosis/Mucorale (unspecified)
Mekonnen et al. (2021), United States [31]	Uncontrolled diabetes, asthma, hypertension, hyperlipidemia, steroid for COVID	Yes	Yes	Yes	Definite	Right globe proptosis, edema of the eyelids, and chemosis of the conjunctiva. right maxillary, ethmoid, and frontal sinuses heavily obscured	Rhino-orbital mucormycosis
Monte Junior et al. (2020), Brazil [32]	Uncontrolled diabetes, steroid for COVID	Yes	Yes	Yes	Definite	Stomach ulcers, intense anemia, fever, acute diarrhea, blood in stool	Gastrointestinal mucormycosis/Mucorale (unspecified)
Moorthy et al. (2021), India [33]	Uncontrolled diabetes (n = 6), steroid for COVID (n = 16)	-	Yes (n = 16)	Yes	Definite	Cellulitis of the face, maxillary sinusitis, headache, necrosis of the palate bone or mucosa, and sudden blindness	Rhino-orbital (n = 6), rhino-orbital-cerebral (n = 5), rhino-cerebral (n = 3), sinusitis alone (n = 3), rhino-orbital (n = 6), Mucorale (unspecified)
Pasero et al. (2020), Italy [34]	Hypertension, lymphopenia	Yes	No	Yes	Putative	Cavitary lesions in the left lung with fluid accumulation in pleural space, pulmonary infiltrates, increased parenchymal thickness of the entire left lung, and left maxillary sinus cloudiness	Pulmonary mucormycosis
Pauli et al. (2021), Brazil [10]	Uncontrolled diabetes	-	No	Yes	Definite	Ulcerated lesion with coagulative necrosis, hemorrhage	Palatal ulcer/ Mucorale (unspecified)

**Table 3:** Treatment outcomes Table showing the treatment and prognosis of mucormycosis in COVID-19 patients.

Author, year	Time between COVID diagnosis and mucormycosis (days)	Treatment (surgery)	Antifungal therapy/treatment	Prognosis
Alekseyev et al. (2021) [14]	-	Yes	Amphotericin B	Survived
Bellanger et al. (2021) [21]	15	-	Amphotericin B	Died
Dallalzadeh et al. (2021) [22]	6	No	Amphotericin B, isavuconazole	Died (n = 2)
Garg et al. (2021) [23]	17	Planned for right lobe lobectomy	Amphotericin B	Survived
Hanley et al. (2020) [24]	-	No	No	Died
Johnson et al. (2021) [25]	-	-	Amphotericin B, voriconazole	Discharged
Kanwar et al. (2021) [26]	16	Yes	Amphotericin B	Died
Karimi‐Galougahi et al. (2021) [27]	21	Yes	Systemic antifungals	Survived
Khatri et al. (2021) [28]	90	Yes	Amphotericin B, posaconazole	Died
Maini et al. (2021) [29]	18	Yes	Amphotericin B, fluconazole	Survived
Mehta et al. (2020) [30]	10	Yes	Amphotericin B	Died
Mekonnen et al. (2021) [31]	7	Yes	Amphotericin B, caspofungin, etc.	Died
Monte Junior et al. (2020) [32]	5	No	No	Died
Moorthy et al. (2021) [33]	-	Yes (n = 7)	Amphotericin B	Survived (n = 11), died (n = 6), follow-up cannot be done (n = 1)
Pasero et al. (2020) [34]	17	No	Amphotericin B, isavuconazole	Died
Pauli et al. (2021) [10]	8	Yes	Amphotericin B	Survived
